# Supplementary material for: Plasmon-driven synthesis of individual metal@semiconductor core@shell nanoparticles
Source: Nat Commun. 2020 Aug 7;11:3957. doi: 10.1038/s41467-020-17789-y (PMC7414885; doi:10.1038/s41467-020-17789-y)
Supplement: Supplementary file 1 — Supplementary Information [file 41467_2020_17789_MOESM1_ESM.pdf]

Supplementary Information

Plasmon-driven synthesis of individual metal@semiconductor core@shell nanoparticles

Kamarudheen et al.

Contents

Supplementary Note 1: Ensemble Au@CeO_2 core@shell nanoparticle synthesis. 3

Supplementary Note 2: Characterization of Au nanospheres. 4

Supplementary Note 3: Temperature increase on Au nanospheres under 532 nm illumination. 5

Supplementary Note 4: Temperature increase on Au nanoparticles as a function of laser intensity. 6

Supplementary Note 5: Color change of Au nanoparticles after CeO_2 shell growth. 7

Supplementary Note 6: Scattering cross-section of Au@CeO_2 core@shell nanoparticles. 8

Supplementary Note 7: Control experiments. 9

- Supplementary Note 7.1: Absence of shell growth in dark corroborated by electron-microscopy. 9
- Supplementary Note 7.2: Absence of shell growth in dark corroborated by optical microscopy. 10
- Supplementary Note 7.3: Stability of nanoparticles under laser irradiation. 10

Supplementary Note 8: Hot-hole contribution to Au@CeO_2 core@shell growth. 12

Supplementary Note 9: Effective thermal conductivity of the surrounding media. 13

Supplementary Note 10: Absorption cross-section with increasing ceria shell thicknesses. 15

Supplementary Note 11: Using ΔPL to estimate the plasmon resonance. 16

Supplementary Note 12: Estimating shell thickness, absorption cross-section and temperature from PL measurements. 20

Supplementary Note 13: CeO_2 shell growth on Au nanorods. 21

Supplementary Note 14: Absorption cross-section of Au nanorods. 22

Supplementary Note 15: Mie resonances in Au@ZnO core@shell nanospheres 23

Supplementary Note 16: Needle-like formation of ZnO nanoparticles in ensemble conditions. 24

Supplementary Note 17: Photoluminescence of Au@ZnO and Au@ZnS core@shell nanoparticles. 25

Supplementary Note 18: Resolution for substrate patterning. 27

Supplementary References 28

Supplementary Note 1: Ensemble Au@CeO_2 core@shell nanoparticle synthesis.

The Au@CeO_2 core@shell nanoparticle synthesis is slow at room temperature, while leading to a CeO_2 shell thickness of 10 nm upon heating to 90 °C for 1 h. We confirm the growth of ceria shell around the Au nanoparticles at high temperatures by observing a red-shift of the plasmon resonance in extinction spectroscopy. Furthermore, we utilize electron microscopy and energy dispersive X-ray characterization to visualize the formed ceria shell.

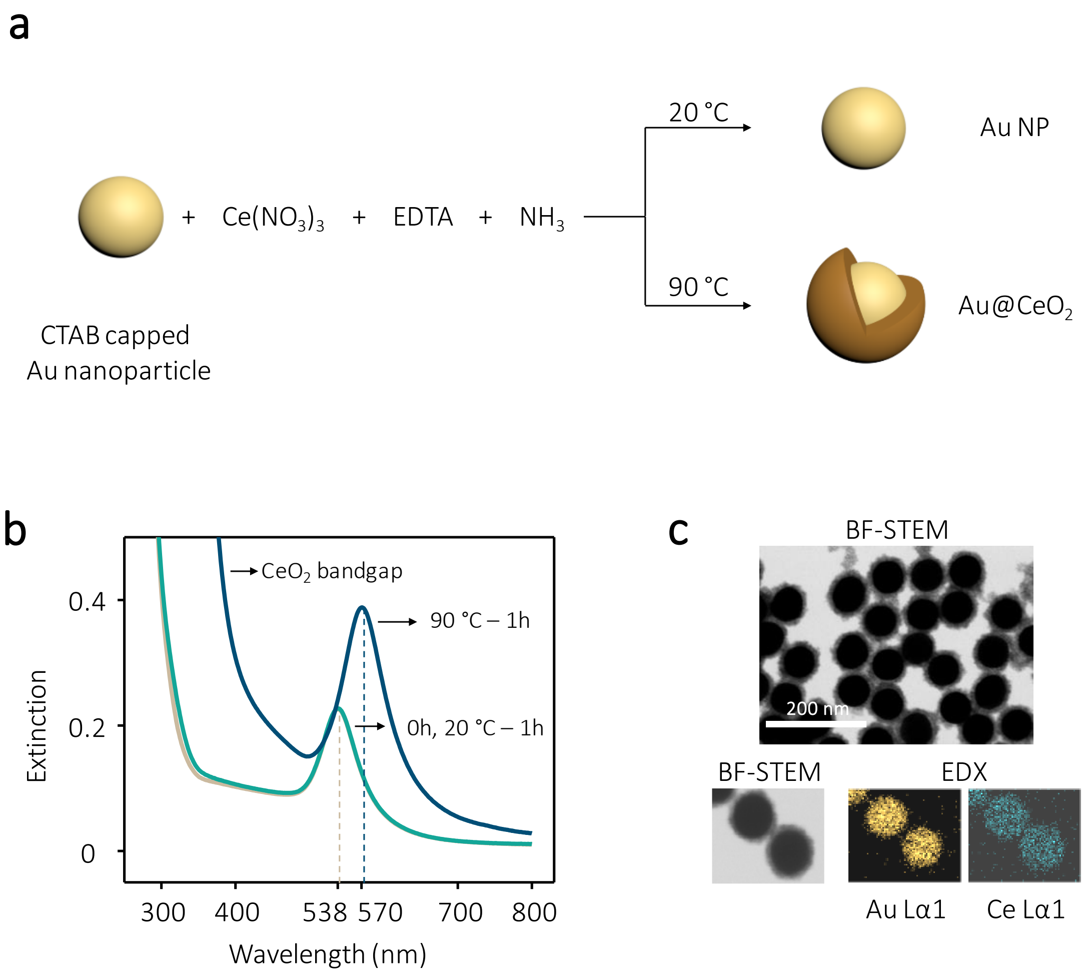

Supplementary Fig. 1: a, Schematic representation of the temperature-activated synthesis of Au@CeO_2 core@shell nanoparticles[1]. **b**, Ensemble extinction spectra of Au nanoparticles mixed with the Ce^{3+}-EDTA solution, kept at room temperature at time = 0 h (brown), time =1 h (green), and when heated to 90 °C using a conventional hot plate for 1 h (blue). **c**, Bright field scanning transmission electron micrograph (BF-STEM) and Au Lα1 (brown) and Ce Lα1 (blue) energy dispersive X-ray (EDX) maps of Au@CeO_2 core@shell nanoparticles synthesized at 90 °C using a conventional hot plate for 1 h.

Supplementary Note 2: Characterization of Au nanospheres.

Au nanoparticles of 66 nm are synthesized colloidally using a seed-mediated method, for both ensemble and photothermal shell growth experiments. TEM images indicate the formation of monodisperse Au nanospheres.

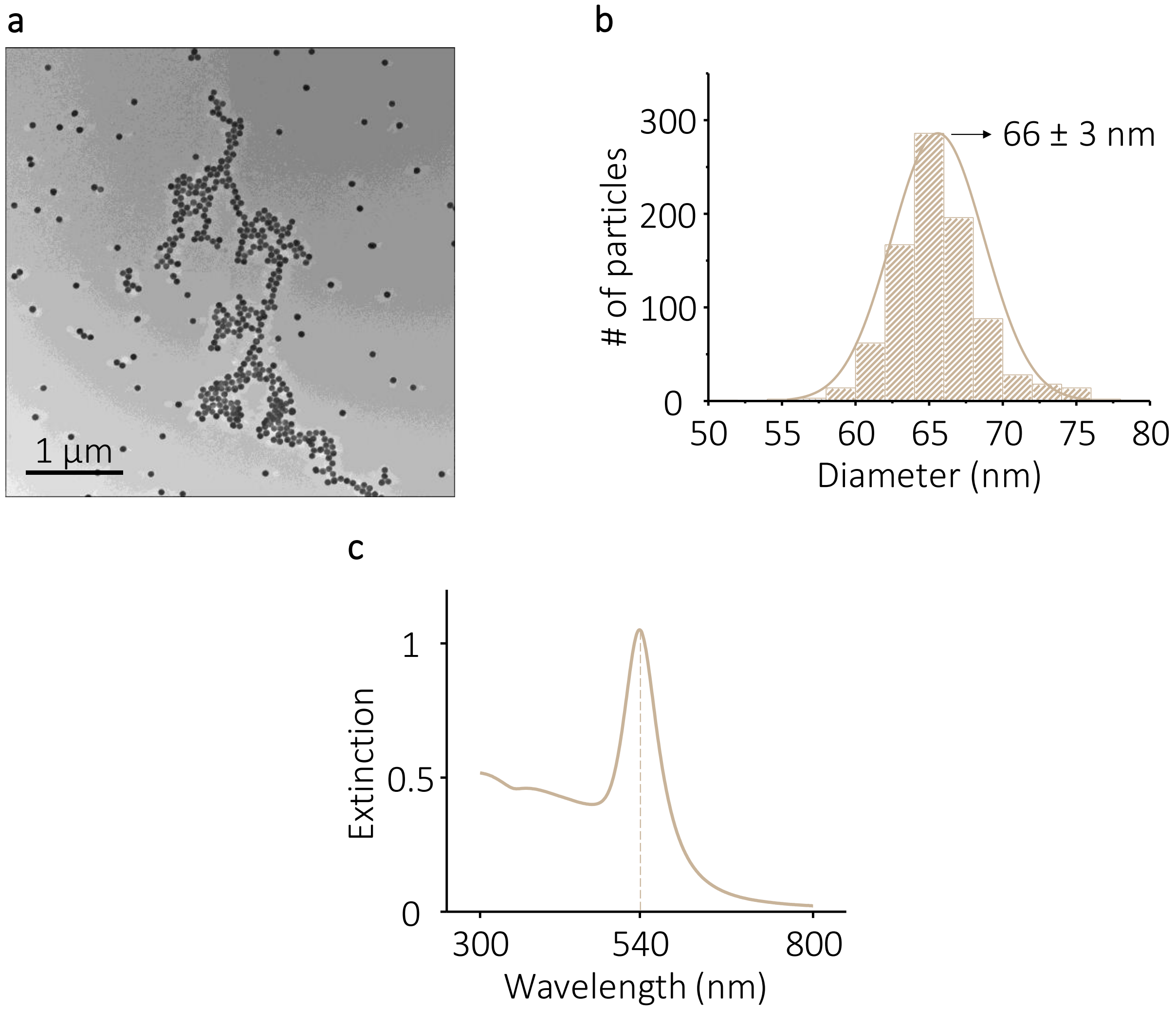

Supplementary Fig. 2: a, Representative TEM image of colloidally synthesized Au nanospheres. **b**, Size-distribution analysis of Au nanospheres obtained from ImageJ software, indicating a mean size of 66 nm. 883 particles are used for analysis. **c**, Extinction spectrum of a colloidal suspension of Au nanoparticles, showing an LSPR at 540 nm.

Supplementary Note 3: Temperature increase on Au nanospheres under 532 nm illumination.

The temperature increase on a nanoparticle surface under laser illumination is dictated by the absorption cross-section and the radius of the nanoparticle, for a fixed laser intensity.

Under a 532 nm continuous wave laser irradiation, Au nanospheres in water generate the highest temperature when they possess a diameter of 79 nm. The Au nanospheres we synthesize have a diameter of 66 nm, which can generate 92% of the temperature generated for 79 nm particles, at any given intensity (See also Eq 1 in main text).

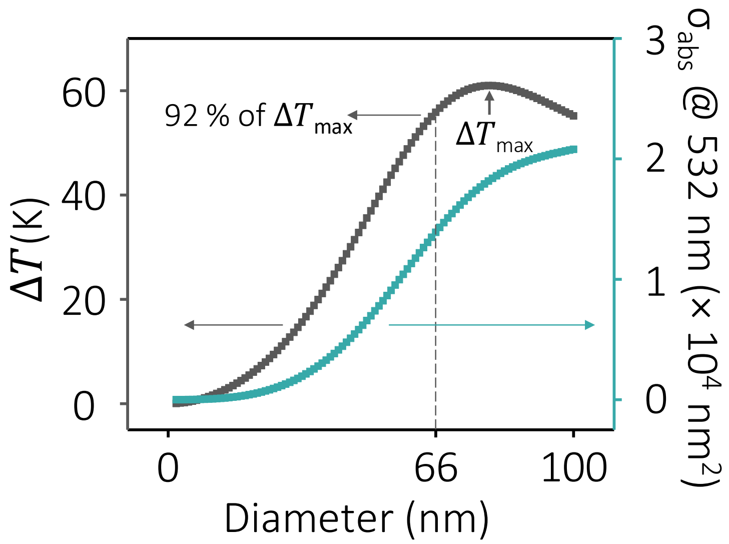

Supplementary Fig. 3: Size dependence of the absorption cross-section of Au nanospheres in water at 532 nm (right axis, teal) and corresponding nanoparticle temperature increase (left axis, gray), for a power density of 1 mW/μm^2.

Supplementary Note 4: Temperature increase on Au nanoparticles as a function of laser intensity.

From equation 1 in the main text, the temperature increase on a nanoparticle is dependent on the illumination intensity. We vary the laser intensity to tune the nanoparticle surface temperature, which in turn dictates the size of the photothermally grown core@shell nanoparticles.

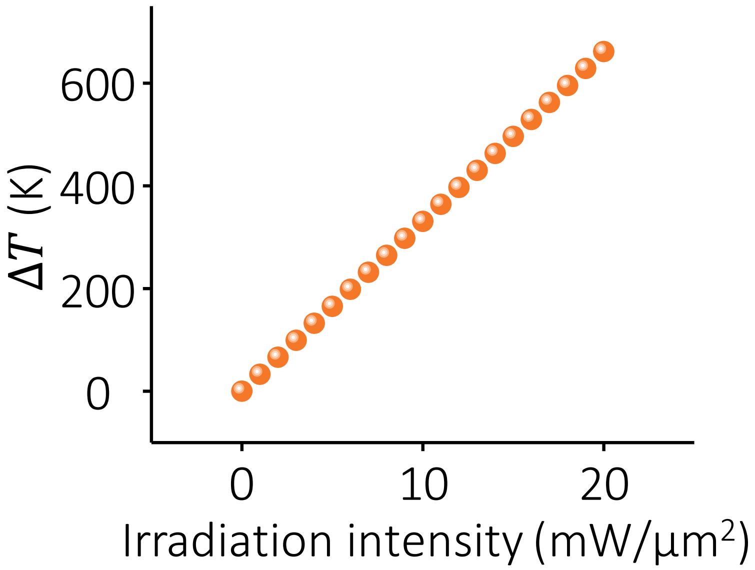

Supplementary Fig. 4: The photothermal temperature increase on the nanoparticle surface with respect to the ambient temperature is plotted as a function of the applied laser intensity (See also Eq 1 from main text). Here, we use an absorption cross-section of 1.37×10^{-14} m^2 corresponding to 66 nm Au nanoparticles and an average thermal conductivity of 1 W/m/K (κ_{water} = 0.6 and κ_{quartz} = 1.4 W/m/K). Note, the temperature dependence of the thermal conductivity is not considered in these calculations.

Supplementary Note 5: Color change of Au nanoparticles after CeO_2 shell growth.

Au nanoparticles deposited on a quart substrate appear as bright green scatterers, when observed under a dark-field microscope. After photothermal growth of a dielectric shell on these nanoparticles, their plasmon resonance red-shifts, thereby changing the color of these green scatterer to yellowish red.

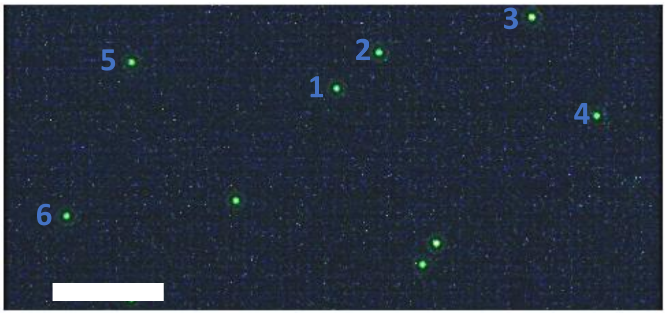

Before irradiation

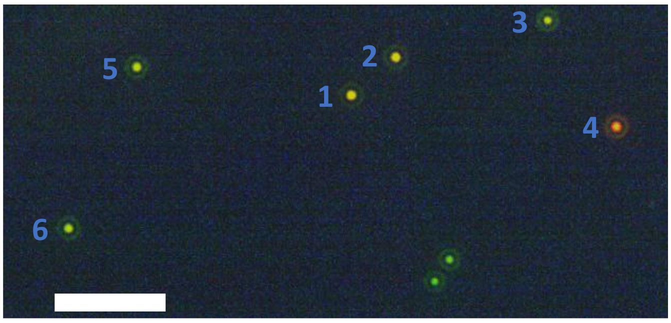

After irradiation

Supplementary Fig. 5: (Top) Dark-field images of Au nanospheres deposited on a quartz substrate before the photothermal growth of CeO_2 shell. These plasmonic particles are green in color. The particles that are numbered are used to grow CeO_2 shell photothermally. Here, one particle is irradiated at a time. (Bottom) Dark-field images of photothermally grown Au@CeO_2 core@shell nanospheres. Scalebars correspond to 10 µm.

These hierarchical structures after irradiation display scattering in yellow and red, depending on the shift in LSPR. The magnitude of LSPR shift is dependent on the thickness of the grown ceria shell. We vary the thickness of the shell by varying the laser intensity. The numbered particles are irradiated using different laser intensities. For example, particle 1,2 and 3 are irradiated at a power density of ~1.3 mW/µm^2, particle 4 is irradiated at 5 mW/µm^2 and particles 5 and 6 are irradiated at ~1.2 mW/µm^2.

Supplementary Note 6: Scattering cross-section of Au@CeO_2 core@shell nanoparticles.

As a dielectric shell is grown around Au nanospheres, their LSPR red-shifts with a simultaneous increase in its scattering cross-section. Thus, in our single-particle experiments, by estimating the size from the measured LSPR of the initial Au nanoparticle before irradiation and by measuring the LSPR red-shift after the shell growth, one can estimate the thickness of the grown semiconducting shell.

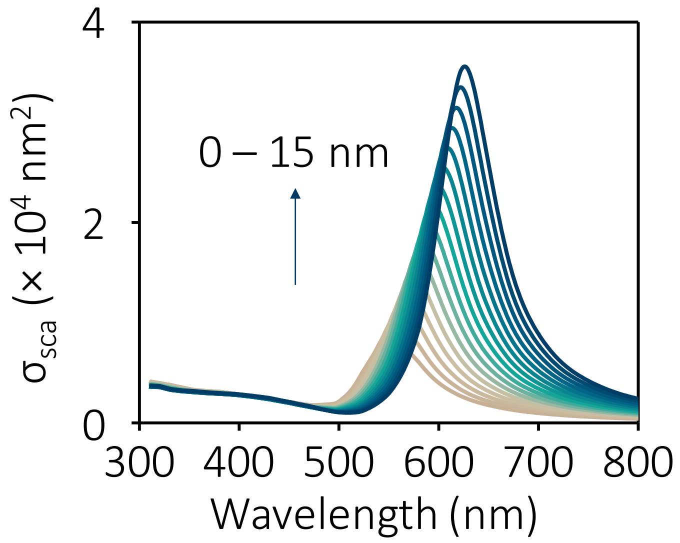

Supplementary Fig. 6: Mie calculations[2] of scattering cross-sections of Au@CeO_2 core@shell nanoparticles, plotted as a function of shell thicknesses ranging from 0 – 20 nm. For the calculations, we consider 66 nm Au nanoparticles with a ceria shell, which are suspended in water. The refractive index of CeO_2 and water used for these calculations are 2.3 and 1.33 respectively.

Supplementary Note 7: Control experiments.

We perform several control experiments to attribute the growth of ceria shell around Au nanoparticles to photothermal heating.

- **Supplementary Note 7.1: Absence of shell growth in dark corroborated by electron-microscopy.**

In our studies, we confirm the growth of a ceria shell under photothermal heating by performing electron microscopy imaging of the laser irradiated nanoparticles. These nanoparticles are deposited on a SiN TEM membrane. From TEM measurements, we also find that the particles away from the illumination spot do not exhibit any ceria shell growth, despite having the same chemical environment as that of the irradiated particle in Fig. 1D of the main text.

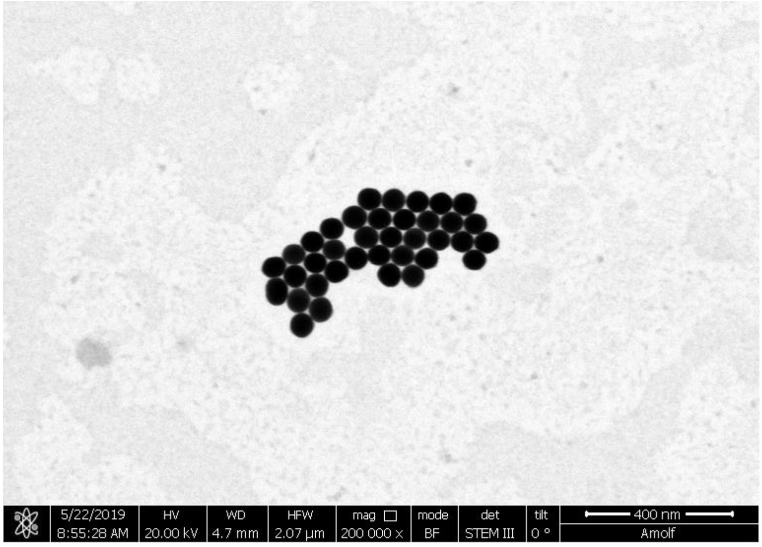

Supplementary Fig. 7.1: BF-STEM image of Au nanoparticles deposited on a silicon nitride membrane, which are away from the irradiation spot.

- **Supplementary Note 7.2: Absence of shell growth in dark corroborated by optical microscopy.**

In Figure 7.2 we plot the scattering spectra of Au nanoparticles adjacent (> 5 µm) to a nanoparticle that is irradiated, before and after laser irradiation. The LSPR of the Au nanoparticles that are not irradiated remains unchanged after flowing the ceria growth solution for 30 min, confirming no changes to the nanoparticle surface and no ceria shell growth.

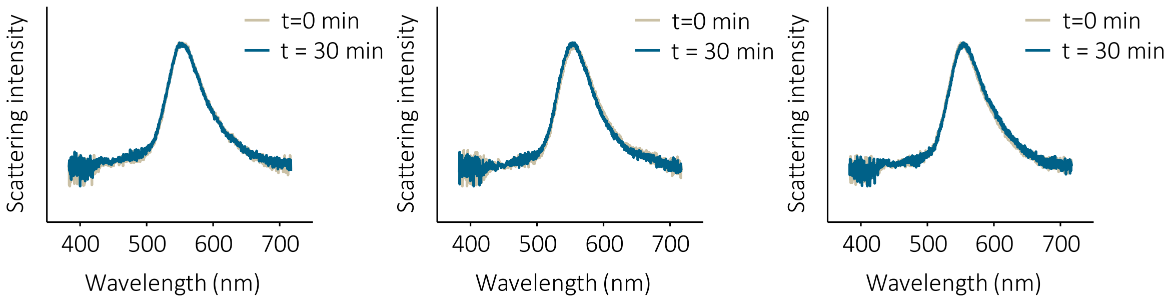

Supplementary Fig. 7.2: Dark-field scattering spectra of three different single Au nanospheres measured, while flowing a solution of Ce^{3+} ions and EDTA at times t = 0 min and t = 30 min in the absence of laser irradiation.

- **Supplementary Note 7.3: Stability of nanoparticles under laser irradiation.**

A solution consisting of CTAB and EDTA-NH_3 mixture is flown over the Au nanospheres deposited on a quartz window of a flow cell. In the absence of Ce^{3+} ions, no spectral changes are observed even under laser irradiation for 15 min. The unchanged spectra before and after irradiation rules out the possibility of any nanoparticle deformation under laser intensities of our choice.

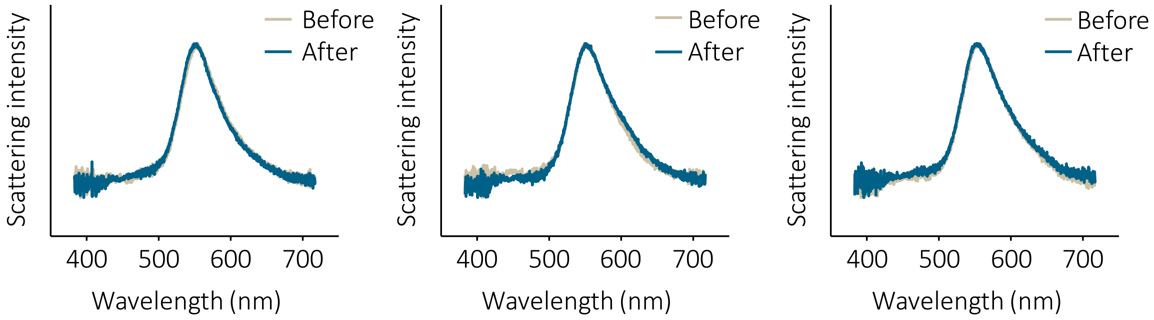

Supplementary Fig. 7.3: Dark-field scattering spectra of three different single Au nanospheres before and after laser irradiation, in absence of Ce^{3+} ions.

Supplementary Note 8: Hot-hole contribution to Au@CeO_2 core@shell growth.

The potential contribution of hot-holes generated under plasmon excitation in driving the Au@CeO_2 core@shell nanoparticles is analyzed, by correlating the Fermi level of Au and the band diagram of CeO_2[1]. Since, we use a 532 nm irradiation laser, which corresponds to an energy of 2.33 eV, the highest energy that a hole could attain is -7.6 eV with respect to the vacuum level. Since the valence band of CeO_2 lies at -7.8 eV with respect to the vacuum level, the holes would not be able to diffuse to the ceria shell under plasmon excitation using 532 nm laser. Moreover, the Schottky nature of the Au-CeO_2 interface would block any possibility of holes being transported to the semiconductor. Also, such holes are generated in the d-band of Au through interband excitation, which is relatively flat in nature. As such, these holes are generally considered to possess low velocities and their mean free path length is considered to be less than 5 nm[3]. These considerations safely allow us to rule out any significant contribution of hot holes to the plasmon-driven synthesis of Au@CeO_2 core@shell nanoparticles.

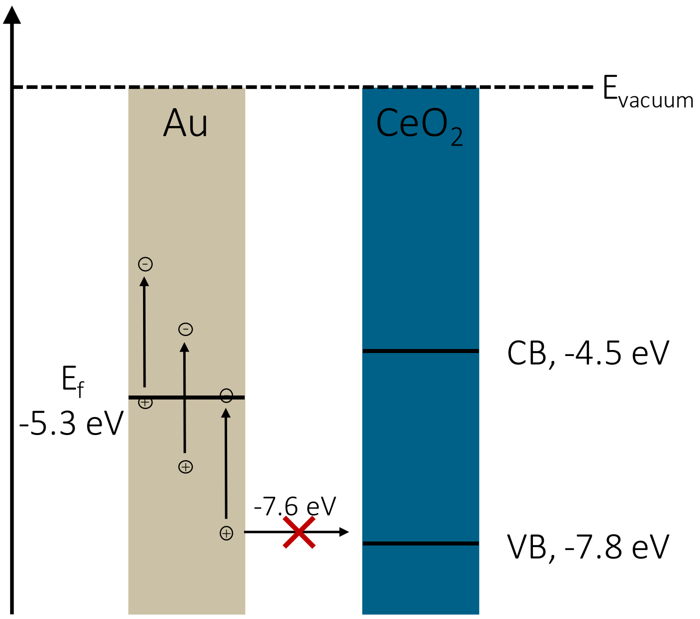

Supplementary Fig. 8: Correlating the Fermi level (E_f) of Au with the conduction band (CB) and the valence band (VB) of CeO_2, by aligning the vacuum energy levels of both materials. The arrows on Au represents the optical transitions under 532 nm excitation.

Supplementary Note 9: Effective thermal conductivity of the surrounding media.

In our experiments, the Au nanoparticles are located at the quartz-water interface. To calculate the temperature increases on the nanoparticle under laser irradiation, a precise knowledge on the thermal conductivity of the surroundings is essential, according to equation 1 in main text. Here, we plot the effective thermal conductivity around the nanoparticle at different temperatures.

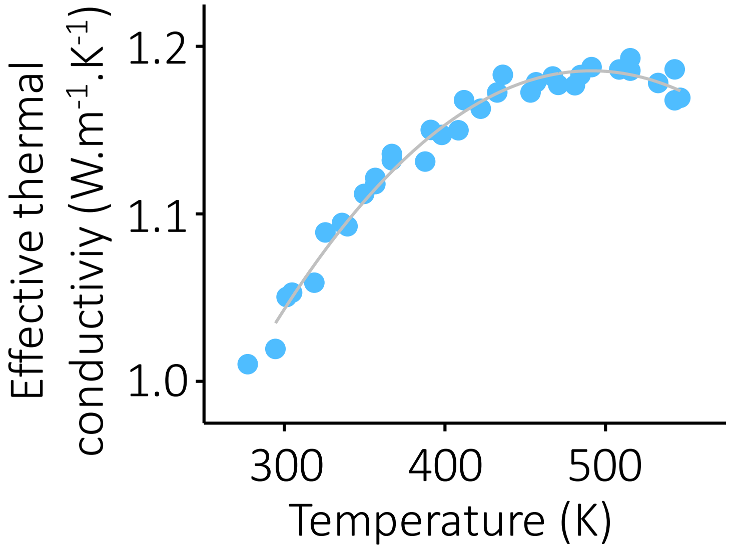

Supplementary Fig. 9: Effective thermal conductivity of the surrounding media of Au nanoparticles constituting water and quartz is plotted as a function of temperature.

In order to calculate the effective thermal conductivity, we take the mean thermal conductivity of water (0.6 W/m/K at 293 K) and quartz (1.4 W/m/K at 293 K). The data for the thermal conductivity of quartz substrate is obtained from the manufacturer website. We also take into account the varying thermal conductivity with temperature, as plotted in Supplementary Fig. 9. The grey line denotes the quadratic fitting of the data points (R^2 = 0.977), according to:

$$y = 0.24544 + 0.00383x - 3.89671 * 10^{-6}x^2$$

We then calculate the nanoparticle surface temperature T, using the above quadratic equation and Eq 2 (main text), to obtain:

$$(T - 293) * (0.24544 + 0.00383T - 3.89671 * 10^{-6}T^2) = \frac{\sigma_{abs}P}{8\pi^2 R\sigma_l^2}\exp\left(-\frac{\left(x - x_p\right)^2 + \left(y - y_p\right)^2}{2\sigma_l^2}\right)$$

Supplementary Note 10: Absorption cross-section with increasing ceria shell thicknesses.

In our photothermal growth experiments, we always use a 532 nm cw laser. As the ceria shell grows in size, the plasmon resonance starts to red-shift. As such the absorption cross-section of the nanoparticle at the illumination wavelength decreases. As such, the nanoparticle temperature also decreases according to equation 1, leading to slower shell growth over the illumination period.

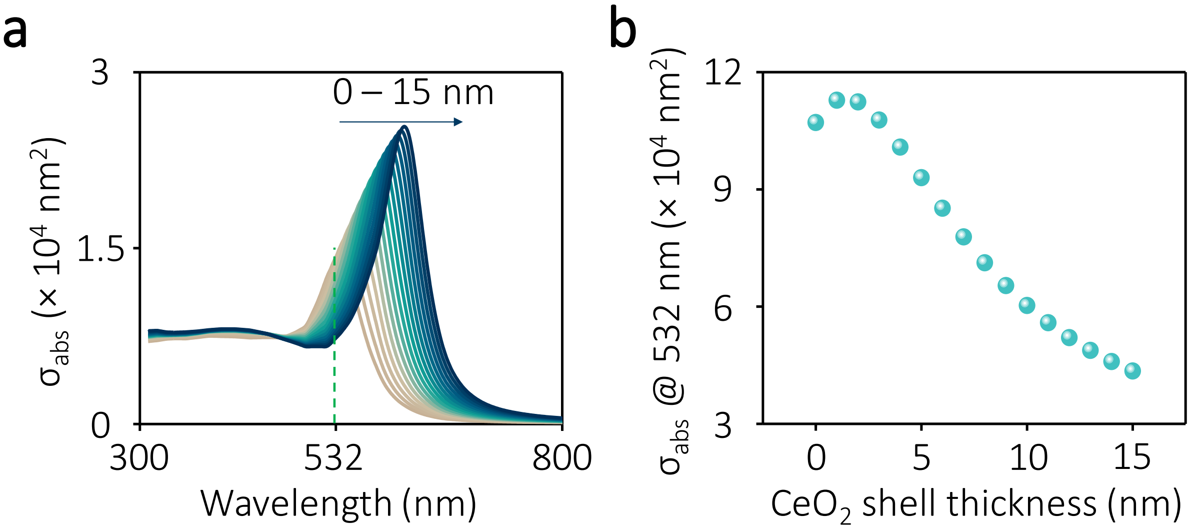

Supplementary Fig. 10: a, Absorption cross-section of Au@CeO_2 core@shell nanospheres, calculated using Mie theory for shell thicknesses ranging from 0 – 15 nm. **b**, Absorption cross-section of Au@CeO_2 nanospheres at 532 nm (corresponding to our laser irradiation wavelength) plotted as a function of ceria shell thickness. In these calculations, we consider Au nanoparticles of 66 nm in diameter suspended in water.

Supplementary Note 11: Using ΔPL to estimate the plasmon resonance.

In this section, we show how ΔPL can be a good proxy for the LSPR of the growing core@shell nanoparticle under laser irradiation.

In our experiments, we measure the inelastic Stokes scattering under nanoparticle illumination, to extract the core@shell growth kinetics (Supplementary Fig. 11a). To obtain ΔPL, we subtract inelastic emission signals at time t, with the signal at t = 0 (Supplementary Fig. 11b). Using this approach, we remove background contributions from the underlying quartz substrate and the (negligible) Raman signal of reactants, and isolate the photoluminescence of the plasmonic nanoparticle. As stated in the main text, the photoluminescence of metallic nanoparticles is proportional to their photonic density of states, corresponding to their scattering spectra. Our ΔPL approach generates a curve that is equivalent to the differential scattering spectrum, where the scattering cross-section at time t (Au@CeO_2), is subtracted with the scattering spectra at t = 0 (Au).

At large shell thicknesses, where the plasmon resonance is red-shifted, the maxima of the ΔPL matches exactly with the plasmon resonance of the core@shell nanoparticle (Supplementary Fig. 11c). At smaller shell thicknesses, a red-shift the maximum of ΔPL compared to the plasmon resonance is expected (Supplementary Fig. 11d). This deviation can be accounted by correlating the LSPR maximum against the maximum of the differential spectrum (Supplementary Fig. 11e). In principle, such approximations should allow us to properly estimate the reaction kinetics under irradiation. In reality, accurately estimating the plasmon resonance under irradiation is more challenging, due to photothermal heating of the surrounding medium and multiple photon absorption, as discussed below (and briefly in the main text).

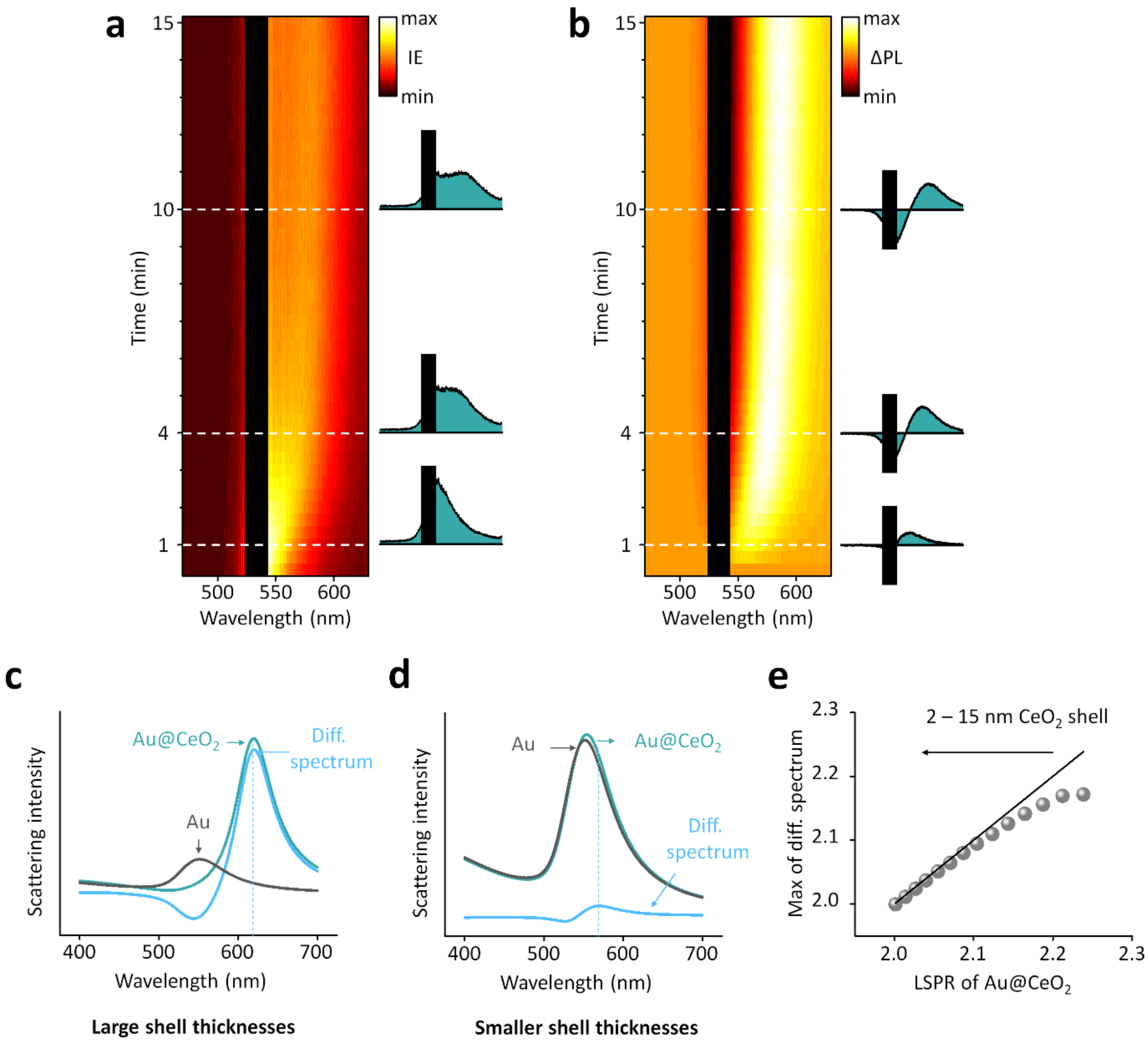

Supplementary Fig. 11: (a,b), Full colormap of the time evolution of the inelastic emission (IE) spectra (**a**) and ΔPL spectra (**b**) measured over a period of 15 min, corresponding to the data in Fig. 3a,b (main text). **c**, FDTD simulations of scattering cross-sections of 62 nm diameter Au (gray line) and corresponding Au@CeO_2 core@shell (teal) nanoparticles with 15 nm shell thickness on a quartz substrate immersed in water. The blue line corresponds to the spectral difference of the scattering cross-sections of Au@CeO_2 and Au nanoparticle, which is henceforth referred as the differential spectrum. The maximum of differential spectrum correlates well to the maximum of Au@CeO_2 scattering cross-section. **d**, FDTD simulations of scattering cross-sections of 62 nm diameter Au (gray line) and corresponding Au@CeO_2 core@shell (teal) nanoparticles with 2 nm shell thickness on a quartz substrate immersed in water. The blue line corresponds to the spectral difference of the scattering cross-sections of Au@CeO_2 and Au nanoparticle. The maximum of differential spectrum here displays a red-shift compared to the plasmon resonance of the core@shell nanoparticle. **e**, Correlation of the maximum of the differential spectrum against the corresponding LSPR of Au@CeO_2 core@shell nanoparticles (gray dots), at various ceria shell thicknesses ranging from 2 – 15 nm. The black straight line denotes a 1 : 1 correlation.

In our experiments, we find a blue-shift for the max[ΔPL(t)] with respect to their LSPR after irradiation (Fig. 3c in main text), which can be attributed to two factors: 1) large nanoparticle surface temperatures, which can **decrease the refractive index of the surrounding medium**, which in turn blue-shifts the plasmon resonance and therefore the particle's PL, and 2) high laser intensities that lead to **successive photon absorption** inside the metal nanoparticle, that can move electron distributions to higher energy levels.

1. **Decreasing refractive index of surrounding medium**: The high temperatures generated on the surface of the nanoparticles can change the refractive index of the surrounding water medium. This lowering of refractive index of the surrounding medium, leads to a blue-shift of the plasmon resonance. Since the photoluminescence of a nanoparticle spectrally follows its plasmon resonance, the decreasing refractive index of surrounding medium at high temperatures leads to a blue-shift of the PL. Mie theory calculations for a 66 nm diameter Au nanoparticle shows that the plasmon resonance blue-shifts by ~15 meV when the particle is heated from room temperature (293 K, refractive index of water - 1.333) to 220 °C (493 K, refractive index of water - 1.293).

2. **Successive photon absorption:** Previously, Link *et al.* have reported a blue-shift in the inelastic photoluminescence of Au nanorods with respect to their corresponding elastic dark-field scattering spectra[4]. They reported a blue-shift of 16-23 meV, depending on the interband excitation wavelength. They argue that such blue-shift is attributed to the higher energy of hot-charge carrier distributions under higher excitation powers. Under such laser intensities, successive photon absorption takes places before the excited hot-charge carriers can relax in to the lattice. As such, the overall energy of the hot carrier distributions is raised, leading to a blue-shift of the photoluminescence spectra. In their experiments, they calculate the average time intervals between successive photon absorption to be between 100 – 500 fs, which

matches the hot-electron thermalization time scales. Furthermore, in their experiments they calculate an increase in surface temperatures of less than 30 K, which rules out nanoparticle reshaping induced blue-shift of photoluminescence. In our experiments, the average time interval between two photon absorption is in the order of 5 fs. Such higher laser intensities in our experiments will also lead to higher energy distributions of hot-charge carriers, which can contribute to blue-shift of the plasmon resonance.

Supplementary Note 12: Estimating shell thickness, absorption cross-section and temperature from PL measurements.

By plotting the max[ΔPL] as a function of time for the growing core@shell nanoparticle, we can estimate the absorption cross-section, diameter, and *ΔT* of the growing nanoparticle as a function of time, by correlating them with FDTD calculations. Such insights can be useful to study the kinetics of nanoparticle syntheses and also to design novel nanoparticle syntheses.

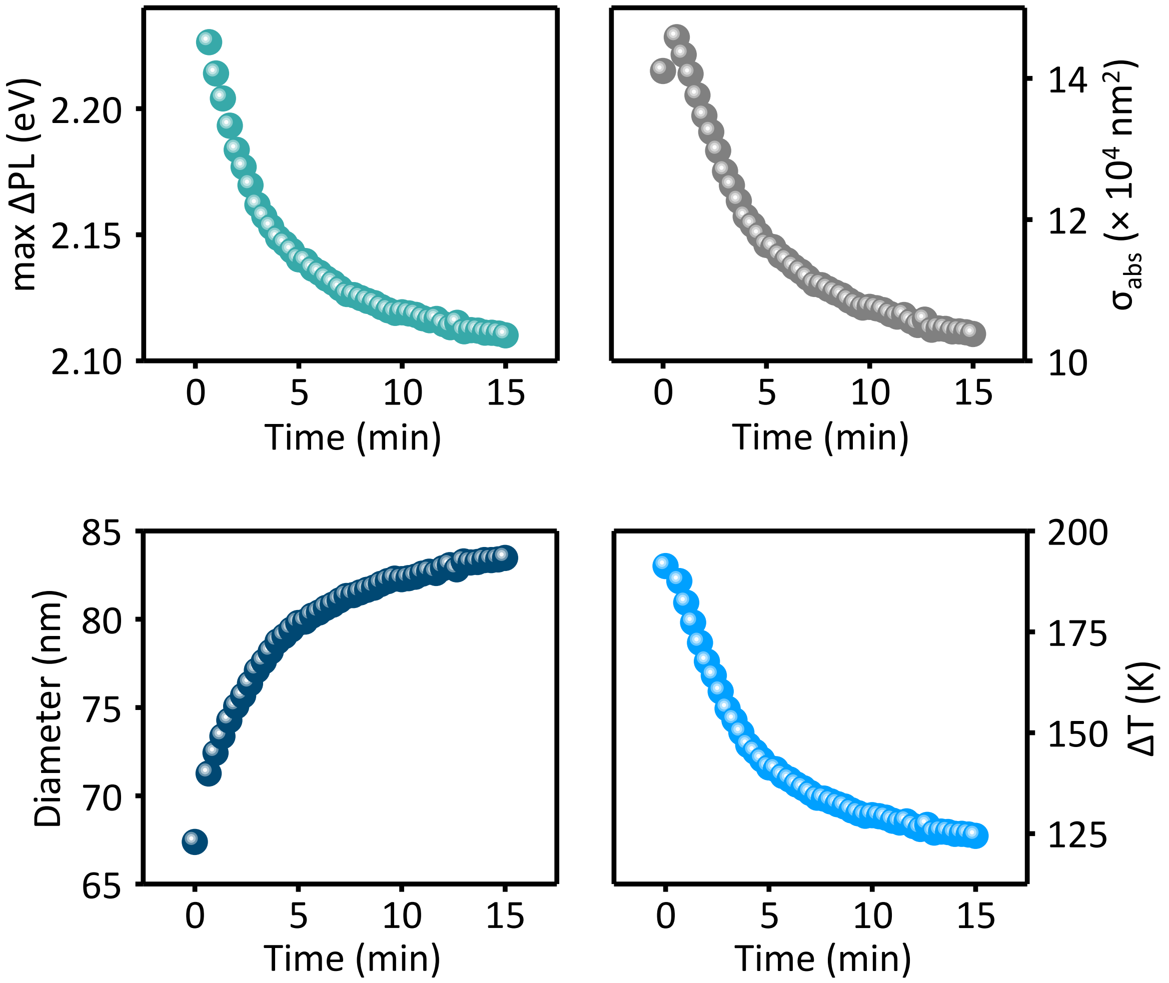

Supplementary Fig. 12: From the inelastic scattering spectra shown in Fig 3**a-b** (main text), we can estimate the plasmon resonance (teal curve, top left), absorption cross-section (gray curve, top right), diameter (navy blue curve, bottom left) and surface temperature (sky blue, bottom right) of the growing nanoparticle using FDTD calculations and equation 2.

Supplementary Note 13: CeO_2 shell growth on Au nanorods.

Using FDTD simulations, we can estimate the photothermal ceria shell growth on Au nanorods. By measuring the LSPR of the Au nanorods before and after irradiation, we can calculate the red-shift in the plasmon resonance, which can then be used to estimate the ceria shell thickness by assuming a constant refractive index of ceria.

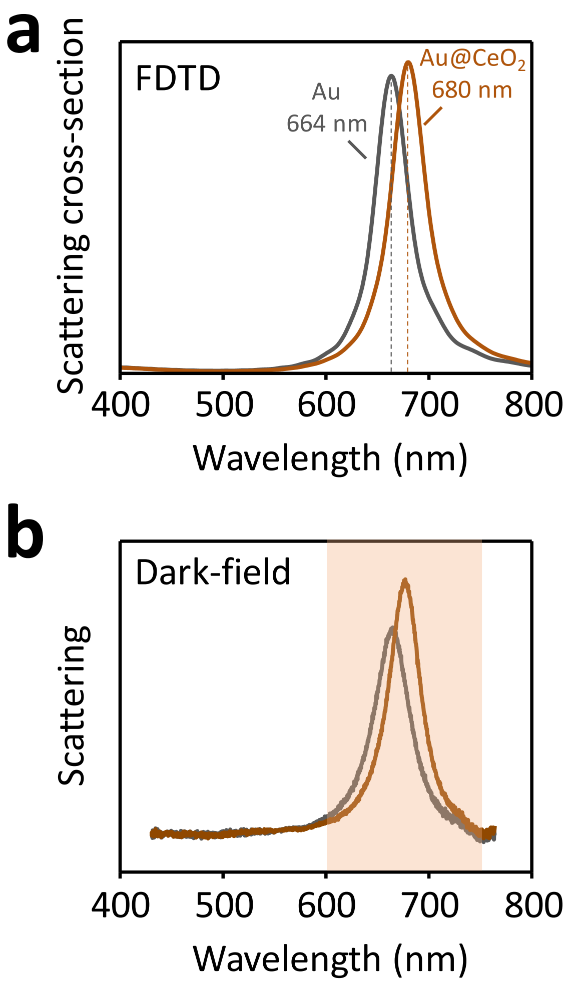

Supplementary Fig. 13: (**a**) FDTD simulation of the scattering cross-sections of a 25 × 59 nm gold nanorod (gray) and a $Au@CeO_2$ core@shell nanorod (orange) with 0.75 nm shell thickness. A 16 nm LSPR red-shift is observed for the growth of 0.75 nm shell thickness. In both the calculations, the nanorods are considered to be at the quartz-water interface. (**b**) Experimentally measured dark-field scattering spectra of Au and $Au@CeO_2$ core@shell nanoparticles corresponding to the data in Fig. 4b (main text). The orange shaded area denotes the spectral range shown in Fig. 4b (main text).

Supplementary Note 14: Absorption cross-section of Au nanorods.

Nanorods possess two different resonances namely transverse and longitudinal corresponding to the short axis and the long axis respectively. Using our 532 nm cw laser, we excite the short axis or the transverse resonance to photothermally grow a ceria shell.

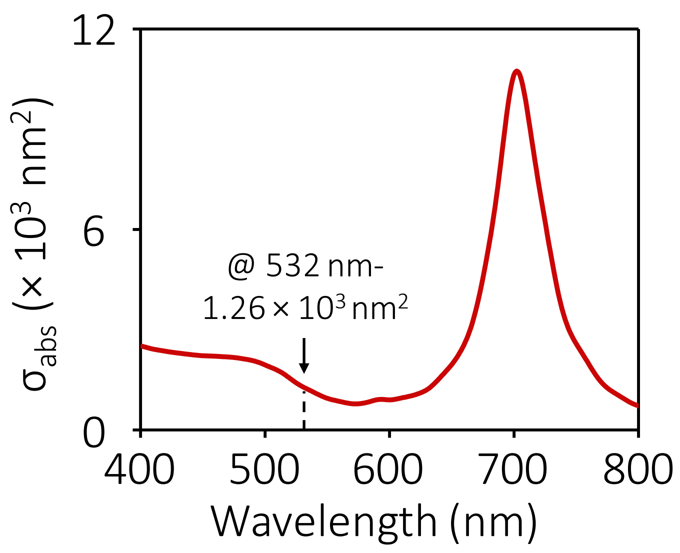

Supplementary Fig. 14: Calculated absorption cross-section of Au nanorods with a dimension of 78 × 25 nm in water using FDTD.

The absorption cross-section of these rods at 532 nm is less than that of Au nanospheres, by roughly an order of magnitude. As such, we expect a much lower photothermal temperature increase on these nanorods, which explains the measured small ceria shell thickness.

Supplementary Note 15: Mie resonances in Au@ZnO core@shell nanospheres

The growth of a thick dielectric shell (>100 nm) around Au nanoparticles lead to the generation of bright particles, which have multiple resonances. These resonances are known as geometric Mie resonances. When we grow a ZnO shell over Au nanoparticles, we find the appearance of these Mie resonances, indicating a large ZnO shell thickness.

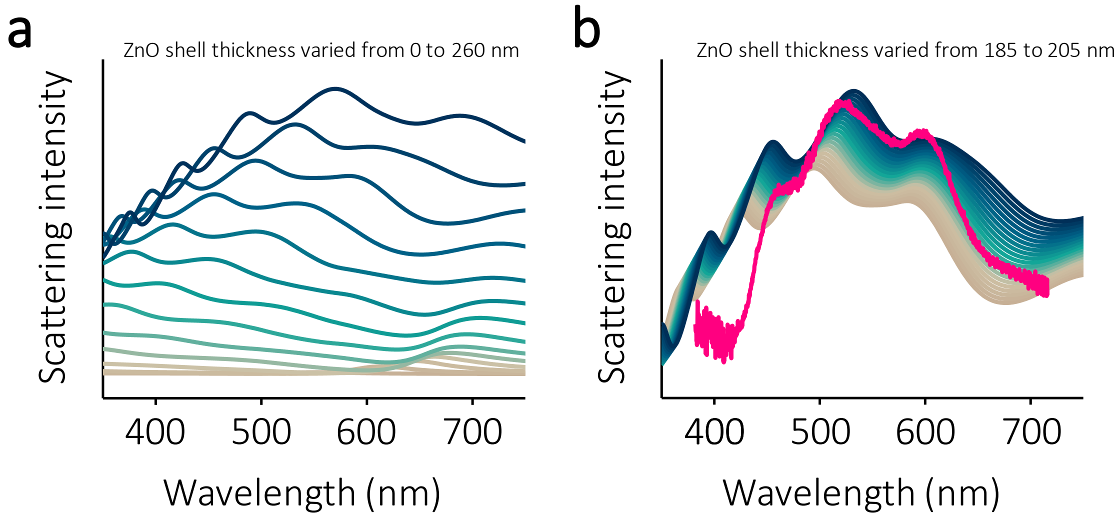

Supplementary Fig. 15: (Top) Mie calculations of scattering cross-sections of Au@ZnO core@shell nanospheres, where ZnO shell thickness is varied between 0 – 260 nm in steps of 20 nm (brown to blue curve). In this calculation, an Au nanoparticle of 70 nm diameter is considered. As the shell thickness is increased, multiple resonances start appearing in the scattering spectra, which is attributed to their morphological Mie resonances. (Bottom) The scattering cross-section of Au@ZnO core@shell nanospheres is plotted, where ZnO shell thickness is varied from 185 – 205 nm (brown to blue curve). The measured spectrum of a photothermally grown Au@ZnO core@shell nanoparticle (pink spectrum) is overlapped with the Mie calculated core@shell spectra.

Supplementary Note 16: Needle-like formation of ZnO nanoparticles in ensemble conditions.

We find that at high temperatures, ZnO forms needle-like structures which are micron-sized, as reported previously. Au nanospheres are observed to be adsorbed on the surface of these ZnO needles. Using the same reaction procedure, we photothermally grow ZnO spheres around Au nanospheres and observe isotropic growth of ZnO shells using polarization-dependent measurements. Such isotropic growth under photothermal synthesis is drastically different from ensemble-synthesis, displaying how nanoscale temperature gradients can be used to accurately shape nanoparticles.

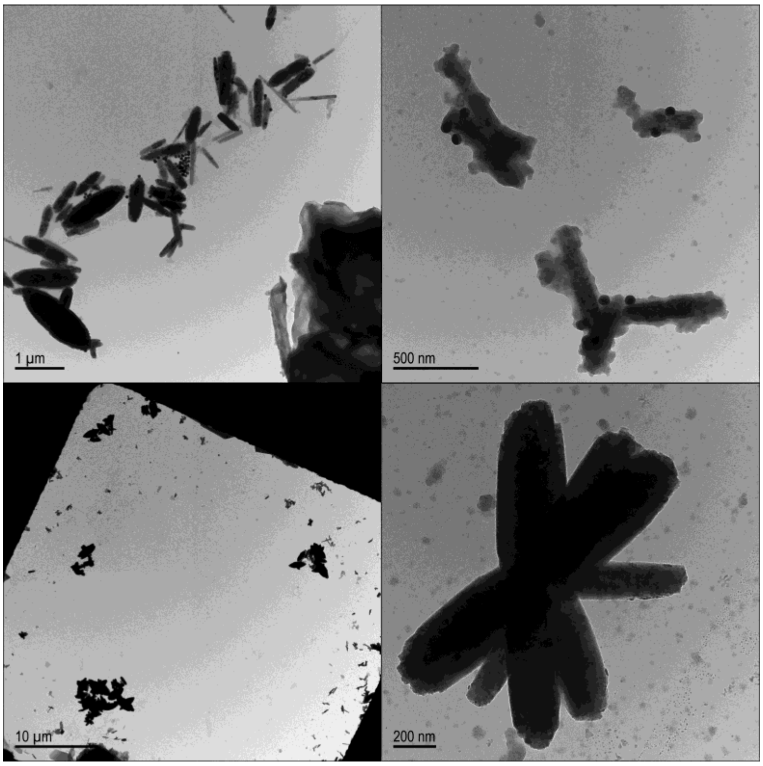

Supplementary Fig. 16: TEM images of ensemble-grown Au-ZnO nanoparticles at 90 °C.

Supplementary Note 17: Photoluminescence of Au@ZnO and Au@ZnS core@shell nanoparticles.

Tracking the photothermal growth of ZnO and ZnS is challenging due to the intrinsic photoluminescence of these semiconductors.

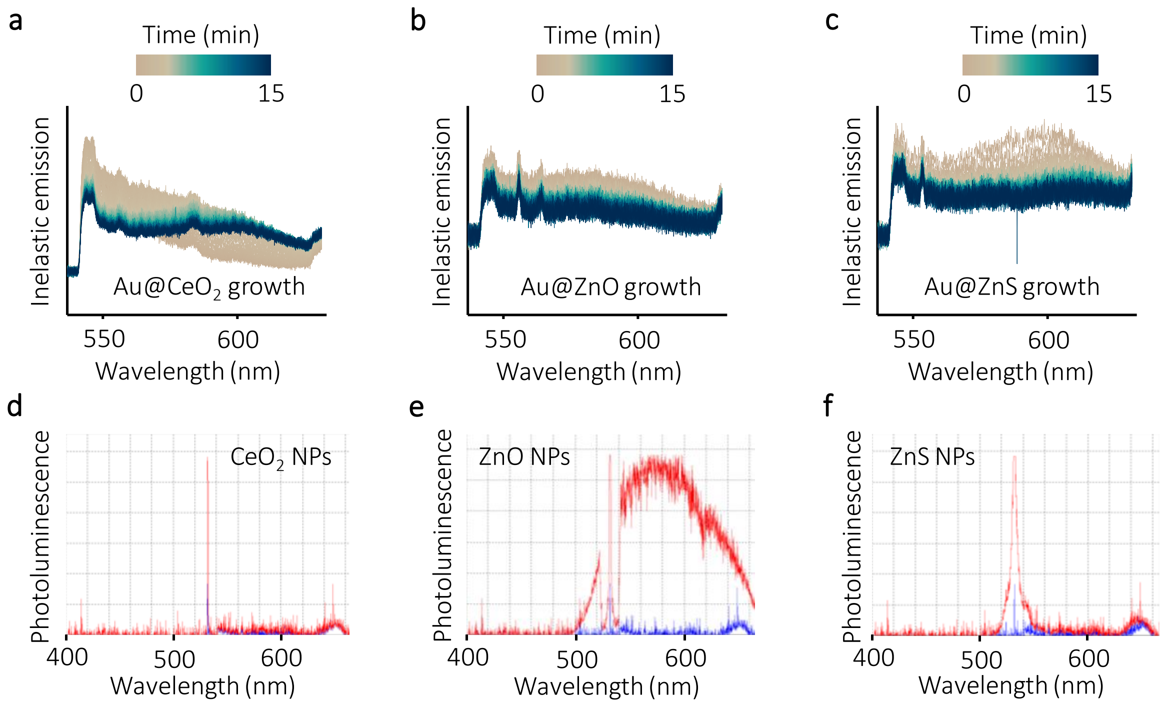

Supplementary Fig. 17: a-c, Representative inelastic scattering spectra obtained over a period of 15 minutes of illumination, during the photothermal growth of a single (**a**) Au@CeO_2, (**b**) Au@ZnO, (**c**) Au@ZnS core@shell nanosphere. **d-f**, Ensemble photoluminescence spectra of CeO_2 (**d**, red curve), ZnO (**e**, red curve) and ZnS (**f**, red curve) nanoparticle suspensions in water are plotted, under a 532 nm irradiation. The blue spectra in panels (**d-f**) correspond to the inelastic scattering signal of water.

The photothermal growth of CeO_2 can be followed by using our ΔPL approach, while in the case of ZnO and ZnS, it is challenging to track the growth as the inelastic emission intensity decreases with increasing time. The decrease in emission intensity indicates non-radiative quenching of the PL of the Au nanoparticle by the growing ZnO and ZnS semiconductor.

Since the bandgap of ZnO and ZnS are above the illumination energy, the luminescence exhibited from these materials should originate from the defects present in them. We postulate that defects in ZnO

and ZnS could quench the photoluminescence of Au nanoparticles, preventing the *in situ* tracking of growth kinetics in photothermal experiments. In case of CeO_2 nanoparticles, the lack of any optically active defects (Panel **d**) allows us to *in situ* track their photothermal growth over Au nanoparticles.

Supplementary Note 18: Resolution for substrate patterning.

By tuning the reaction conditions, illumination parameter such as the laser power and spot size and the size and the interparticle distance of the nanoparticle array, one can localize chemical reactions on to a single nanoparticle without activating any reactions on the neighboring particles.

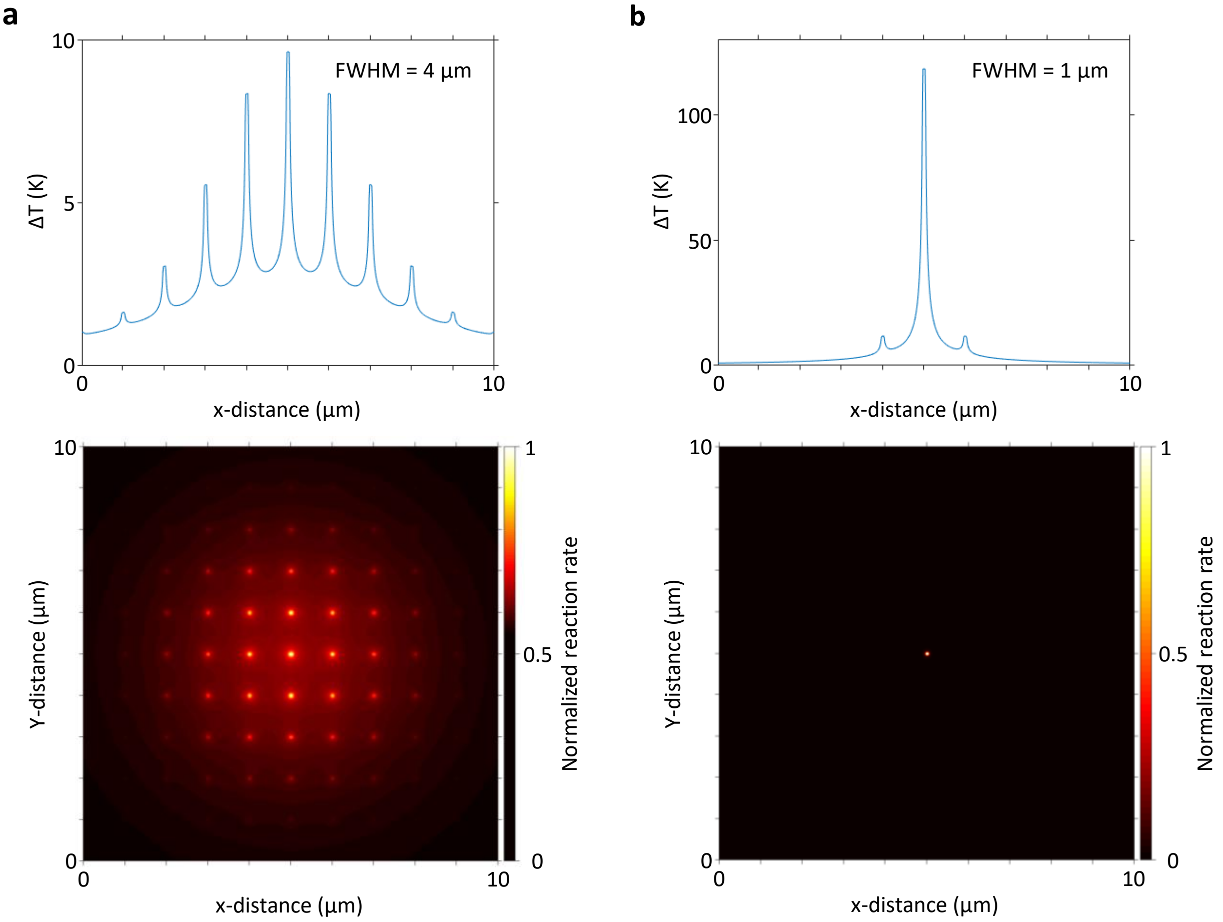

Supplementary Fig. 18: a, (Top) Cross-cut of calculated temperature increase on an array of 66 nm Au nanoparticles with an interparticle spacing of 1 µm, under a 532 nm Gaussian illumination of 4 mW power and FWHM of 4 µm, adapted from Fig.5 **l**-left in main text. (Bottom) Colormap of photothermal rate enhancement of a chemical reaction of activation energy of 50 kJ/mol, on the array of nanoparticles corresponding to temperature increases in Fig. 5 **l**-left in main text. **b**, Similar cross-cuts of calculated temperature and photothermal rate enhancement for the same array and illumination conditions, except when the FWHM is changed to 1 µm. The particle at the centre of the Gaussian beam in panel (**b**) displays a 166 times increase in the chemical reactivity compared to its adjacent nanoparticle.

Supplementary References

1. Li, B. *et al.* (Gold Core)@(Ceria Shell) Nanostructures for Plasmon-Enhanced Catalytic Reactions under Visible Light. *ACS Nano* **8**, 8152–8162 (2014).

2. Mie, G. Beiträge zur Optik trüber Medien, speziell kolloidaler Metallösungen. *Ann. Phys.* **330**, 377–445 (1908).

3. Bernardi, M., Mustafa, J., Neaton, J. B. & Louie, S. G. Theory and computation of hot carriers generated by surface plasmon polaritons in noble metals. *Nat. Commun.* **6**, 7044 (2015).

4. Cai, Y.-Y. *et al.* Photoluminescence of Gold Nanorods: Purcell Effect Enhanced Emission from Hot Carriers. *ACS Nano* **12**, 976–985 (2018).
